# Supplementary material for: Polyanions provide selective control of APC/C interactions with the activator subunit
Source: Nat Commun. 2019 Dec 20;10:5807. doi: 10.1038/s41467-019-13864-1 (PMC6925294; doi:10.1038/s41467-019-13864-1)
Supplement: Supplementary file 1 — Supplementary Information [file 41467_2019_13864_MOESM1_ESM.pdf]

Supplementary Information

Polyanions provide selective control of APC/C interactions with the activator subunit

Arda Mizrak and David O. Morgan

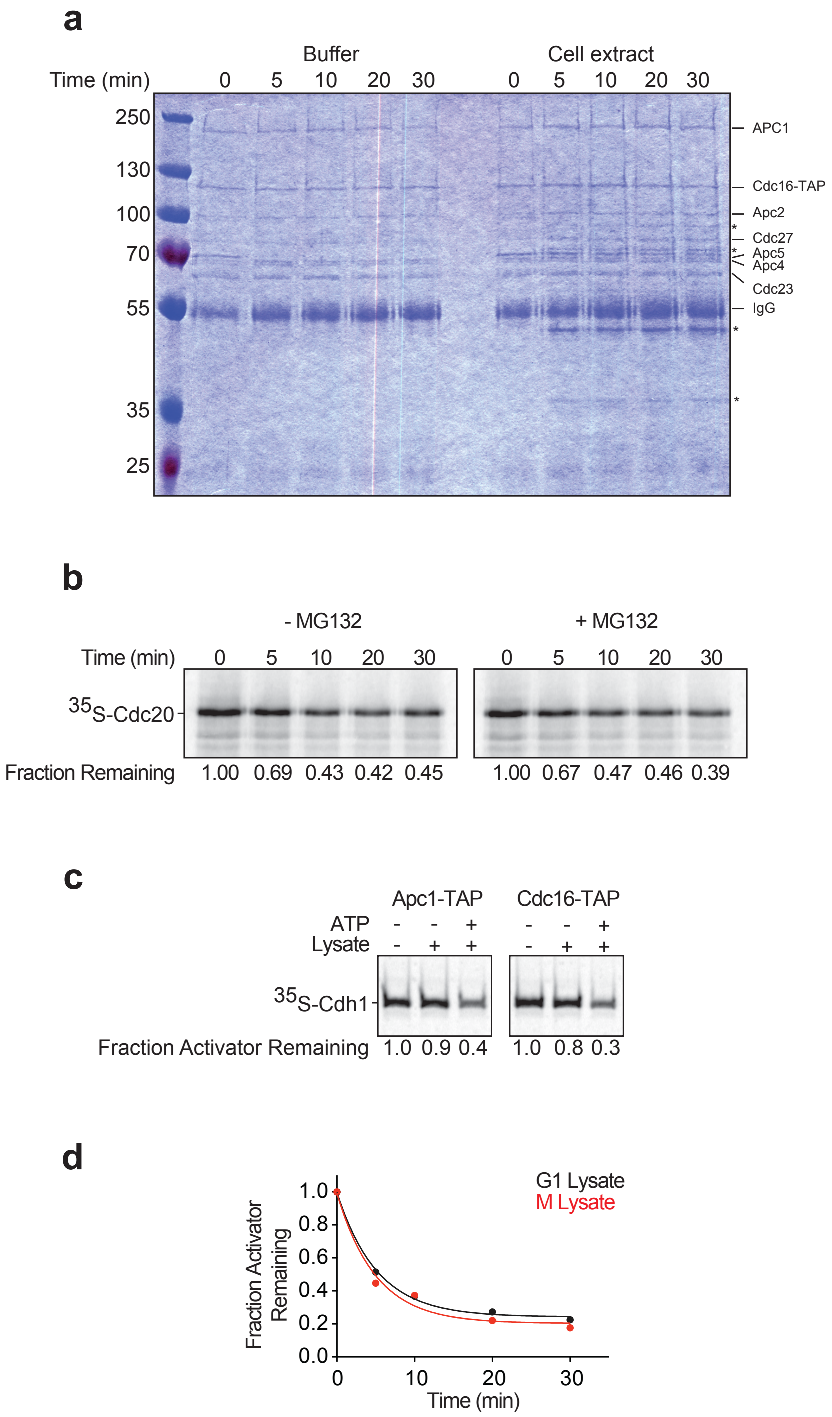

Supplementary Figure 1. Characterization of dissociation activity

a. APC/C subunits remain intact during the activator dissociation reaction. As in Fig. 1c, dissociation reactions were performed with buffer or yeast lysates, terminated at the indicated times, and analyzed by SDS-PAGE and staining with Coomassie Blue. Nonspecific lysate proteins that bind to IgG beads are indicated by asterisks.

b. Cdc20 dissociation reactions were performed with yeast lysate (2.5 mg/ml) in the absence (left) or presence (right) of the proteasome inhibitor MG132 (50 μM) plus 5 mM ATP. Uncropped autoradiograph and source data are provided in the Source Data file.

c. APC/C was immunopurified from lysates of cells carrying TAP-tagged Apc1 (left) or Cdc16 (right), and Cdh1 dissociation reactions were performed with yeast lysate plus 5 mM ATP. Uncropped autoradiograph and source data are provided in the Source Data file.

d. Lysates were prepared from wild-type cells arrested in G1 using α-factor (1 μg/ml) or in metaphase with nocodazole (15 μg/ml) and tested in Cdh1 dissociation assays in the presence of 5 mM ATP. Uncropped autoradiograph and source data are provided in the Source Data file.

a

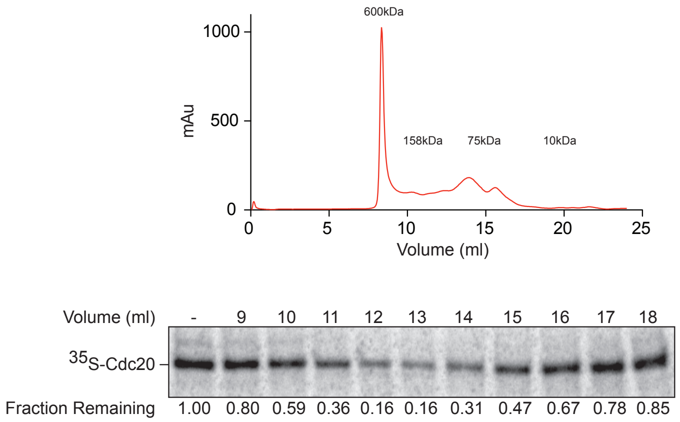

b

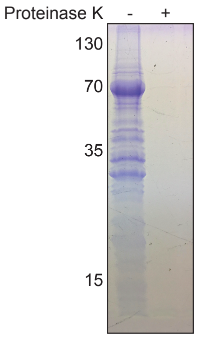

Supplementary Figure 2. Molecular size of dissociation activity

a. Yeast lysates were applied to a DEAE-Fast flow ion exchange column, and dissociation activity was eluted with 500 mM KOAc, concentrated and applied to a Superdex 200 gel filtration column. Top panel shows the UV absorbance (280 nm) of the eluate. In the bottom panel, fractions were concentrated 10-fold and used in a Cdc20 dissociation reaction, supplemented with 5 mM ATP. Uncropped autoradiograph and source data are provided in the Source Data file.

b. As described in Fig. 2d, the hydroxyapatite flow-through fraction from Fig. 2b was treated with buffer (left) or 0.5 U of Proteinase K (right) at 37°C for 1 h, followed by SDS-PAGE and staining with Coomassie Blue. Proteinase K treatment resulted in loss of all visible protein bands. Uncropped gel is provided in the Source Data file.

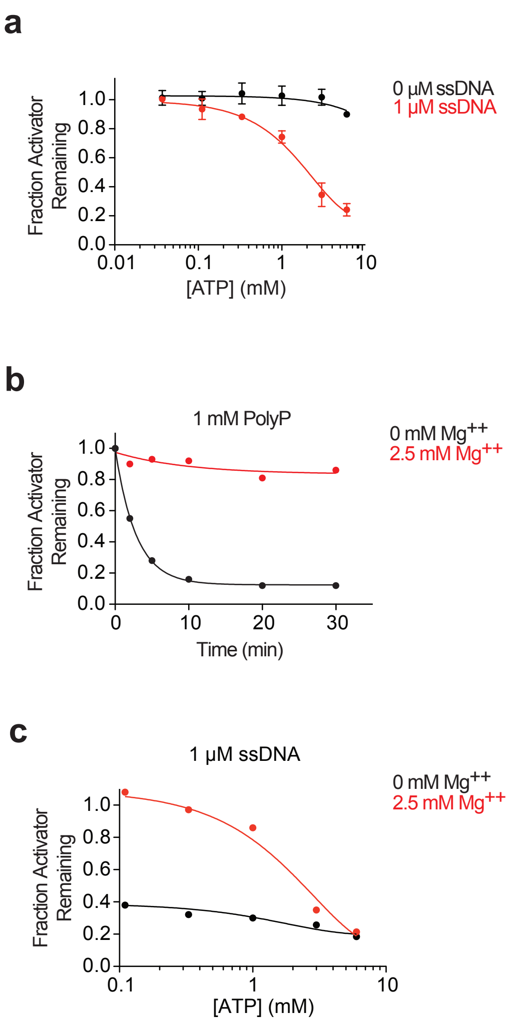

Supplementary Figure 3. ATP stimulates nucleic acid-dependent dissociation by sequestering magnesium ions

a. ATP alone is not sufficient for dissociation. Varying ATP concentrations were tested in Cdh1 dissociation assays. Reactions were performed with buffer alone (black) or 1 µM 75mer ssDNA (red) in the presence of 2.5 mM $MgCl_2$. Data indicate means (+/- SEM) from two independent experiments. Uncropped autoradiographs and source data are provided in the Source Data file.

b. Magnesium inhibits the effects of polyphosphate. Cdh1 dissociation reactions were performed using polyphosphate (1 mM total PO_4^-) in the absence (black) or presence (red) of 2.5 mM $MgCl_2$. Uncropped autoradiograph and source data are provided in the Source Data file.

c. ATP stimulates activator dissociation only when magnesium is present. Cdh1 dissociation reactions were performed using 1 µM 75mer ssDNA. ATP was serially diluted and added to reactions in the absence (black) or presence (red) of 2.5 mM $MgCl_2$. Uncropped autoradiograph and source data are provided in the Source Data file.

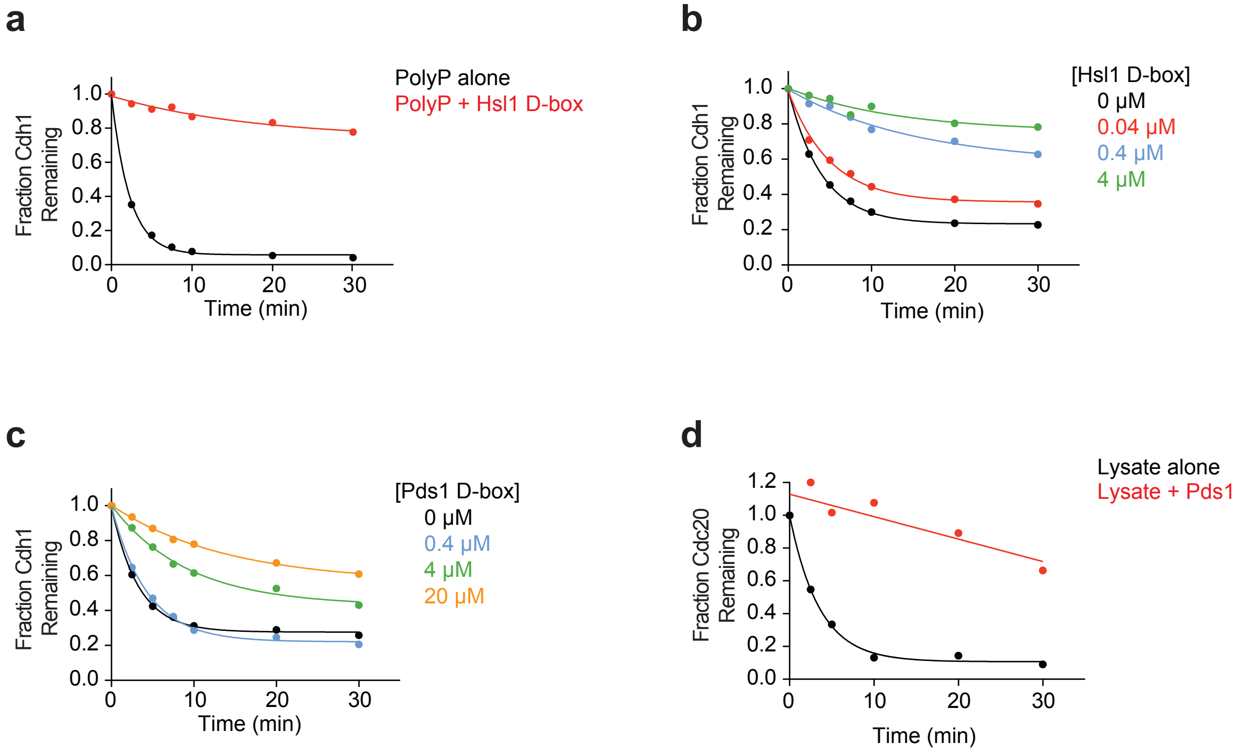

Supplementary Figure 4. D-box binding inhibits activator dissociation

a. Cdh1 dissociation was measured in the presence of polyphosphate (1 mM total PO_4^-) with (red) or without (black) 4 μM Hsl1 D-box peptide. Uncropped autoradiograph and source data are provided in the Source Data file.

b, c. Cdh1 dissociation was measured in the presence of 1.5 μM 75mer ssDNA and varying concentrations of Hsl1 (**b**) or Pds1 (**c**) D-box peptides (black 0 μM, red 0.04 μM, blue 0.4 μM, green 4 μM, orange 20 μM). Reactions were supplemented with 3 mM ATP. Uncropped autoradiographs and source data are provided in the Source Data file.

d. Cdc20 dissociation reactions were performed in the presence of yeast lysate (2.5 mg/ml) and 5 mM ATP, in the absence (black) or presence (red) of 10 μM Pds1 fragment (aa 1-110). His-tagged Pds1 fragment was expressed in bacteria and purified using a nickel column. Uncropped autoradiograph and source data are provided in the Source Data file.

Supplementary Table 1. Nucleic acid and peptide sequences used in this study

T7 antisense sequences were annealed with T7 promoter sequence and used for transcription of the indicated RNAs *in vitro*.

T7-tQ(UUG) antisense	GGAGGTCCCACCCGGATTCGAACTGGGGTTGTTCGGATCAGAACCGAAAG TGATAACCACTACACTATAGGACCTATAGTGAGTCGTATTAATTTC
T7-tS(AGA) antisense	CGACAACTGCAGGACTCGAACCTGCGCGGGCAAAGCCCAAAAGATTTCTAA TCTTTCGCCTTAACCACTCGGCCAAGTTGCCTATAGTGAGTCGTATTAATTTC
T7 random N antisense	NNNNNNNNNNNNNNNNNNNNNNNNNNNNNNNNNNNNNNNNNNNNNNNNNN NNNNNNNNNNNNNNNNNNNNNNNCCTATAGTGAGTCGTATTAATTTC
T7 Promoter	GAAATTAATACGACTCACTATA
25-base ssDNA	AGTTCAGACCAATTGGTAGTTTGTC
50-base ssDNA	TAGGCAGAAGCGGCTACCAAGGTGAATTAATGCGCTTATACACCAATAGG
75-base ssDNA #1	TAGTGACTGGTTTAGCGTCATACCTAGGAGTCCGCACACGGAAACGTGACAT ATCGCTGGATTAACGACCCGAGA
75-base ssDNA #2	TCTCGGGTCGTTAATCCAGCGATATGTCACGTTTCCGTGTGCGGACTCCTAG GTATGACGCTAAACCAGTCACTA

Hsl1 D-box	QNKPKRAALSDITNSFNKMNK-Cy5
Hsl1 D-box^{mut}	QNKPKAAAASDITASFNKMNK-Cy5
Hsl1 KEN box	GVSTNKENEGPEYPTKIEK-Cy5
Pds1 D-box	AQQQGRLPLAAKDNNRSKSFIL-Cy5
