# Supplementary material for: Polyanions provide selective control of APC/C interactions with the activator subunit
Source: Nat Commun. 2019 Dec 20;10:5807. doi: 10.1038/s41467-019-13864-1 (PMC6925294; doi:10.1038/s41467-019-13864-1)
Supplement: Supplementary file 3 — Reporting Summary [file 41467_2019_13864_MOESM3_ESM.pdf]

nature research

Corresponding author(s): David O. Morgan

Last updated by author(s): Nov 27, 2019

Reporting Summary

Nature Research wishes to improve the reproducibility of the work that we publish. This form provides structure for consistency and transparency in reporting. For further information on Nature Research policies, see Authors & Referees and the Editorial Policy Checklist .

Statistics

For all statistical analyses, confirm that the following items are present in the figure legend, table legend, main text, or Methods section.

n/a	Confirmed	
☐	☒	The exact sample size (*n*) for each experimental group/condition, given as a discrete number and unit of measurement
☐	☒	A statement on whether measurements were taken from distinct samples or whether the same sample was measured repeatedly
☒	☐	The statistical test(s) used AND whether they are one- or two-sided<br>*Only common tests should be described solely by name; describe more complex techniques in the Methods section.*
☒	☐	A description of all covariates tested
☒	☐	A description of any assumptions or corrections, such as tests of normality and adjustment for multiple comparisons
☒	☐	A full description of the statistical parameters including central tendency (e.g. means) or other basic estimates (e.g. regression coefficient) AND variation (e.g. standard deviation) or associated estimates of uncertainty (e.g. confidence intervals)
☒	☐	For null hypothesis testing, the test statistic (e.g. *F*, *t*, *r*) with confidence intervals, effect sizes, degrees of freedom and *P* value noted<br>*Give P values as exact values whenever suitable.*
☒	☐	For Bayesian analysis, information on the choice of priors and Markov chain Monte Carlo settings
☒	☐	For hierarchical and complex designs, identification of the appropriate level for tests and full reporting of outcomes
☒	☐	Estimates of effect sizes (e.g. Cohen's *d*, Pearson's *r*), indicating how they were calculated

Our web collection on statistics for biologists contains articles on many of the points above.

Software and code

Policy information about availability of computer code

Data collection	Gel images were acquired using Typhoon 9400 Scanner Control Software v5.
Data analysis	Gel images were quantified with ImageQuant TL (GE Healthcare) and ImageJ (https://imagej.nih.gov). Graphs were generated using Prism 5 (GraphPad). Data points were fitted to exponential single phase decay equation in Prism software.

For manuscripts utilizing custom algorithms or software that are central to the research but not yet described in published literature, software must be made available to editors/reviewers. We strongly encourage code deposition in a community repository (e.g. GitHub). See the Nature Research guidelines for submitting code & software for further information.

Data

Policy information about availability of data

All manuscripts must include a data availability statement. This statement should provide the following information, where applicable:

- Accession codes, unique identifiers, or web links for publicly available datasets
- A list of figures that have associated raw data
- A description of any restrictions on data availability

The source data underlying Figures 1a, c, d, e, 2a, c, d, f, 3a-d, 4a-g, 5a-g, 6a-d, Supplementary Figures 1b-d, 2a, b, 3a-c, and 4a-d are provided as a Source Data file. All other relevant data are available from the authors.

Field-specific reporting

Please select the one below that is the best fit for your research. If you are not sure, read the appropriate sections before making your selection.

☒ Life sciences ☐ Behavioural & social sciences ☐ Ecological, evolutionary & environmental sciences

For a reference copy of the document with all sections, see nature.com/documents/nr-reporting-summary-flat.pdf

Life sciences study design

All studies must disclose on these points even when the disclosure is negative.

Sample size	No statistical methods were used for sample size. Sample sizes of at least 3 different nucleic acid preparations were used throughout the study.
Data exclusions	Quantification of gel images was performed by measuring only the full length translated protein bands. Incomplete translation products were excluded from quantifications.
Replication	All results were replicated in multiple independent experiments.
Randomization	Not applicable
Blinding	Not applicable

Reporting for specific materials, systems and methods

We require information from authors about some types of materials, experimental systems and methods used in many studies. Here, indicate whether each material, system or method listed is relevant to your study. If you are not sure if a list item applies to your research, read the appropriate section before selecting a response.

Materials & experimental systems

n/a	Involved in the study	
☐	☒	Antibodies
☐	☒	Eukaryotic cell lines
☒	☐	Palaeontology
☒	☐	Animals and other organisms
☒	☐	Human research participants
☒	☐	Clinical data

Methods

n/a	Involved in the study	
☒	☐	ChIP-seq
☒	☐	Flow cytometry
☒	☐	MRI-based neuroimaging

Antibodies

Antibodies used	Primary antibodies (polyclonal goat yC-20 (sc- 6731) for Cdc20 and yC-16 (sc-8959) for Cdh1) were purchased from Santa Cruz Biotechnology.
Validation	Primary antibody specificity was confirmed in cells lacking the target protein.

Eukaryotic cell lines

Policy information about cell lines

Cell line source(s)	All yeast strains were derivatives of W303.
Authentication	All strains were confirmed by colony PCR.
Mycoplasma contamination	N/A
Commonly misidentified lines (See ICLAC register)	N/A
